# Exploring the interplay of neuropsychological functions, psychological wellbeing, and lifestyle through principal component analysis: a comprehensive study

**DOI:** 10.3389/fpsyg.2025.1692251

**Published:** 2025-12-18

**Authors:** Nicolas Ayala-Aldana, Marina Ruiz-Rivera, Ariadna Pinar-Martí, Mónica López-Vicente, Oren Contreras-Rodríguez, Jordi Julvez

**Affiliations:** 1 Clinical and Epidemiological Neuroscience (NeuroÈpia), Institut d'Investigació Sanitària Pere Virgili (IISPV), Reus, Spain; 2 Barcelona Institute for Global Health (ISGlobal), Barcelona, Spain; 3 University of Barcelona, Barcelona, Spain; 4 Universitat Pompeu Fabra (UPF), Barcelona, Spain; 5 CIBER Epidemiología y Salud Pública, Instituto de Salud Carlos III, Madrid, Spain; 6 Department of Psychiatry and Forensic Medicine, Autonomous University of Barcelona, Bellaterra, Spain; 7 Centro de Investigación en Red de Salud Mental (CIBERSAM), Instituto de Salud Carlos III, Madrid, Spain; 8 Human Nutrition Unit, Facultat de Medicina i Ciències de la Salut, Universitat Rovira i Virgili, Reus, Spain

**Keywords:** ADHD, adolescence, lifestyle, neuropsychological functions, principal component analysis

## Abstract

**Introduction:**

A balanced psychological wellbeing and healthy lifestyle—which includes regular physical activity and the prevention of alcohol and tobacco use—are relevant for neuropsychological functioning. We aimed to analyze the association between adolescent psychological wellbeing, physical activity, and ever having used alcohol and tobacco with neuropsychological principal components (PC).

**Method:**

This cross-sectional research used the baseline data from a sample of 523 healthy adolescents enrolled in the Walnuts Smart Snack Dietary Intervention Trial from Barcelona. We performed principal components analysis (PCA) to determine neuropsychological PC using working memory, fluid intelligence, emotional recognition, risky decision-making and attention deficit hyperactivity disorder (ADHD) symptoms. Multiple linear regression was used to analyze the relationship between psychological wellbeing, physical activity, and ever used alcohol and tobacco with neuropsychological PC. Regression models were adjusted by child sex, age, maternal education, and socioeconomic status.

**Results:**

Two neuropsychological PC, “ADHD symptoms” and “hot executive function,” were identified in PCA analyses (eigenvalues > 1.0). Psychological wellbeing showed an inverse association with the “ADHD symptoms” PC (β_1_ = −0.04, CI = −0.07, −0.02, *p*-value = < 0.001). Ever having consumed alcohol (β_1_= 0.26, CI = 0.07, 0.44, *p*-value = 0.006) and ever having smoked (β_1_ = 0.66, CI = 0.42, 0.90, *p*-value = < 0.001) were positively associated with “ADHD symptoms” PC. Moderate to vigorous physical activity during leisure time was positively associated with the “hot executive functions” PC (β_1_ = 0.09, 95% CI = 0.00, 0.17, *p*-value = 0.041).

**Conclusion:**

Our main results suggest that less psychological wellbeing, as well as ever having used alcohol or tobacco, was associated with “ADHD symptoms” PC. Physical activity might have a direct association with “hot executive functions” PC in a healthy adolescent sample.

## Introduction

Adolescence is a pivotal stage of preparation for adulthood, characterized by rapid physical, social, and brain development (Larsen and Luna, 2018). The second decade of life is marked by substantial changes in brain structure and high order executive functions, especially in regions and systems associated with response inhibition, risk and reward, and emotion regulation (Steinberg, 2005). Therefore, the development of executive functions—working memory, fluid intelligence and risky decision-making—and emotion recognition are relevant for planning, problem-solving and social relationship in adolescence (Anderson et al., 2001; Davidson et al., 2006). Working memory supports the ability to hold and manipulate information, fluid intelligence enables flexible reasoning in novel situations, and risky decision-making involves evaluating potential rewards and consequences. Emotion recognition, in turn, is essential for interpreting social cues and navigating interpersonal interactions (Spear, 2013). During adolescence, executive functions undergo substantial maturation, paralleling the structural and functional development of the prefrontal cortex (Davidson et al., 2006). These processes can be broadly divided into cold and hot cognition. Cold cognition refers to rational, goal-directed, and emotionally neutral processes, whereas hot cognition involves affectively charged processes such as reward-based decision-making and emotion regulation (Arain et al., 2013). However, during this period, symptoms related to attention-deficit hyperactivity disorder (ADHD) may interfere with the recognition of internal emotions and the execution of some cognitive tasks, such as working memory and decision-making processes, resulting in difficulties in the academic performance (Haza et al., 2024) and social interactions (Ahmed et al., 2022; Li et al., 2020; Hartley and Somerville, 2015). Thus, adolescence is a highly demanding period, psychologically, socially and cognitively speaking (Larsen and Luna, 2018).

Psychological wellbeing and lifestyle play an important role in adolescent brain development and neuropsychological functioning (Viejo et al., 2018; Lima et al., 2022). Psychological wellbeing reflects healthy development, supporting adolescents' optimal functioning and strengthening their mental health (Ryff and Singer, 2008). Previous research identified a longitudinal relationship between cognitive function and wellbeing in a large sample of children and adolescents, revealing that a larger vocabulary in childhood was associated with smaller increases in loneliness over time (Fuhrmann et al., 2021). Likewise, lifestyle is very important to maintain a healthy brain. Indeed, engaging in regular exercise, not smoking, avoid alcohol intake, and getting sufficient sleep are associated with improved mental health (Larsen and Luna, 2018; Sampasa-Kanyinga et al., 2020). Regular physical activity have been associated enhancing executive functions and reducing ADHD symptoms. In turn, alcohol consumption and smoking have been involved in compensation system, negatively affecting learning and working memory functions and increasing ADHD symptoms (Lees et al., 2020; Colyer-Patel et al., 2023). Hence, mental health problems, and inherently psychological wellbeing during adolescence, might be closely related to cognitive function, behavior and lifestyle.

The cornerstone of efforts for the primary prevention of many of the risk factors for neuropsychological impairment is promoting psychological wellbeing and a healthy lifestyle in adolescents (Arafa et al., 2024; Tabrizi et al., 2024). However, several neuropsychological functions are highly correlated (Spear, 2013; Brydges et al., 2017), making it difficult to isolate their pure association with psychological wellbeing or lifestyle. Principal components analysis (PCA), a type of dimensionality reduction technique, addresses the intercorrelation problem and identifies neuropsychological function patterns (Groth et al., 2013). A previous PCA study identified neurocognitive principal component (PC) among the youth population, where higher neurocognitive performance was protective for tobacco consumption (Jones et al., 2024). Overall, studies that do not account for this multicollinearity may report biased results, making it harder to distinguish the specific predictive relationships and potentially attributing an association to a single cognitive domain when several domains are in fact interrelated, whereas examining broader patterns can provide a more comprehensive view of neuropsychological functions. This represents a clear research gap, as understanding these patterns is crucial for accurately characterizing neuropsychological profiles and their associations with behavioral or clinical outcomes.

PCA data-driven analysis on neuropsychological functioning in healthy adolescents and its link to psychological wellbeing and lifestyle is limited. This cross-sectional study aimed to explore these associations, focusing on PCA-derived parameters from neuropsychological functions, including working memory, attention, fluid intelligence, decision-making, emotion recognition, and ADHD-related symptoms, using baseline data from a sample of adolescents participating in the Walnuts Smart Snack Dietary Intervention Trial (WSS). We hypothesized that better psychological wellbeing and a healthy lifestyle (characterized by increased physical activity and non-initiation into alcohol and tobacco consumption) would be independently associated with better neuropsychological performance in the adolescent population.

## Material and methods

### Study design and participants

The goal of the WSS was to determine if adding a dietary supplementation of 30 kernel grams of walnuts per day for 6 months would benefit the neurocognitive and socioemotional development of healthy adolescents (Clinical Trial Identifier: NCT02590848). However, the current study is a cross-sectional investigation using only the WSS baseline data (Julvez et al., 2021).

We enlisted students from 11 evenly distributed high schools in Barcelona over a year (2015–2016). Public and private schools were invited to participate, aiming to include at least one high school per municipal district. Participants completed several neuropsychological tests and provided information on their lifestyle, physical and emotional development. All information about the clinical trial procedure is described in the WSS protocol (Julvez et al., 2021). The original study recruitment included 771 participants, however, only 523 participants who had complete information on the neuropsychological tests were eligible for the present study (Supplementary Figure 1). The study received the board approval from Parc Salut Mar's Clinical Research Ethics Committee (approval number: 2015/6026/I).

### Primary neuropsychological variables

We used seven primary variables prior to conduct the PCA and obtain the neuropsychological PCs.

The computer-based 2-back task was used to assess working memory, where a higher *d* prime (*d*′) score indicates a more accurate performance (López-Vicente et al., 2016). To evaluate fluid intelligence, we administered the computer-based inductive reasoning subtest from the Tests of Primary Mental Abilities (PMA-R) adapted to the Spanish population (Thursthone, 1957). The overall score reflects the number of correct responses, meaning a higher score signifies greater performance. To evaluate risky decision-making, we used the computer-based Roulettes Task. We measured the total risk adjustment index (CUPRAT). The closer to 0 this index is, the less risk sensitivity (Levin et al., 2007). To assess emotion recognition ability, we used the computer-based Benton Emotional Recognition Task (ERT; Montagne et al., 2007). The measures for ERT cover the correct total responses of facial emotions. Thus, higher scores indicate better emotional recognition. The fifth and sixth primary variables were the inattention and hyperactivity scores obtained from the class teachers by responding the Diagnostic and Statistical Manual of Mental Disorders IV (American Psychiatric Association) ADHD form list. For both variables, higher scores indicate more ADHD-related symptoms (American Psychiatric Association, 2000). The last test used was the Strengths and Difficulties Questionnaire (SDQ), a self-reported mental health rating scale. We used the externalizing score of the SDQ, which ranges from 0 to 20 and is calculated by adding the conduct problems and hyperactivity/inatention scales, so a higher score indicates problem behaviors (Goodman, 1997). All the aforementioned tests were administered in its adapted and validated version in Spanish. Further details on the primary neuropsychological variables can be found in Appendix S1. Additionally, internal consistency of ADHD inattention and hyperactivity, as assessed by the SDQ externalizing scale (comprising conduct problems and hyperactivity/inattention), was evaluated using Cronbach's alpha (Supplementary Table 1). Internal consistency for ADHD-related inattention and hyperactivity, as assessed by the SDQ externalizing subscale (including social problems and hyperactivity/inattention), was evaluated using Cronbach's alpha. Neuropsychological development during adolescence encompasses both cognitive and behavioral domains, which are crucial for processing internal and external information, decision-making, and social interactions. To assess neuropsychological functions, we performed a PCA including all variables related to behavioral measures (such as externalizing symptoms and ADHD) and cognitive measures (such as working memory, fluid intelligence, and risky decision-making). These variables have been selected to balance the presence of behavioral and cognitive aspects and to prevent hierarchical components due to over-correlation of neuropsychological variables. For cognition, we used scores of the 2-back task, PMA-R and Roulettes Task. For behavior, we used ADHD hyperactivity symptoms, ADHD inattention symptoms and SDQ externalizing problems. Finally, ERT score was used as an intermediate feature variable (cognition and behavior) in the PCA.

### Reduction of dimensionality and neurocognitive patterns

The seven neuropsychological variables were used to identify the neuropsychological patterns (main outcomes) derived from PCA analysis. PCA is a reduction of dimensionality technique effective for analyzing complex data. The main goal is to reduce the number of variables and detect “intrinsic patterns” in the data based on linear combinations of neuropsychological scores (Groth et al., 2013).

The adequacy of the data for factor analysis was evaluated in advance of the PCA. The Kaiser-Meyer-Olkin test and Bartlett's test of sphericity were conducted before the PCA analysis to observe the relationship between the seven psychometric variables (Supplementary Table 2). PC factors were retained considering the Kaiser criterion to use factors with eigenvalues greater than 1.0 (Kaiser, 1960). The factor structure was then simplified and made easier to understand using PCA with varimax rotation. The rotated component fed the factor loadings for neuropsychological test contained in the factor. To analyze the results, variables with loadings greater than |0.3| were considered to contribute significantly to the pattern. Finally, factor standardized scores were saved for each neuropsychological PC to be used in subsequent multivariate regression analyses. Higher positive standardized scores indicate that participants are strongly represented on the positive variable loadings of the PC, while lower negative standardized scores indicate strong representation on the negative variable loadings of the PC. Finally, bootstrap resampling (*n* = 1,000) and sample splitting (*n* = 3) were performed to assess the reproducibility and stability of the PCs, respectively. Only the unrotated (raw) loadings were compared across bootstrap and resampling iterations because rotation methods (e.g., varimax) are not invariant to resampling and can alter the orientation of the component axes. This makes direct comparison of rotated loadings unstable and unreliable. In contrast, unrotated loadings preserve the original component space, allowing consistent assessment of the reproducibility of the extracted components.

### Psychological wellbeing, lifestyle behavior and sociodemographic characteristics

A fieldwork technician provided psychological and lifestyle questionnaires to the adolescent participants and sociodemographic questionnaires were given to the parents to complete at home and return to us through the school instructors.

Psychological wellbeing was assessed enquiring the adolescents about their mood during the preceding month through the KIDSCREEN-27 questionnaire (Ravens-Sieberer et al., 2007). For lifestyle habits, we inquired about physical activity, alcohol consumption and tobacco use among participants. The frequency of moderate to vigorous physical activity (to the point of being out of breath) outside of school hours was assessed with four multiple-choice options (from “one time or less” to “more than three times” per week). Additionally, participants were asked whether they had ever consumed alcohol, been drunk, used tobacco or used electronic cigarettes. All these variables were recorded in a dichotomous format (yes/no). For tobacco and electronic cigarette use, the variables were combined into a single exposure variable in dichotomous format (yes/no).

Lastly, we collected participant's covariate, including participants' age and biological sex. Anthropometric data were collected by trained nurses using standardized protocols and calibrated equipment (height: SECA 214 stadiometer, weight: SECA 770 scale). The body mass index (BMI), based on the World Health Organization referent, was computed weight (kg)/height (m)^2^ and then converted as *z*-scores (BMI *z*-scores) comparing with reference population. Adherence to the Mediterranean Diet (MedDiet) was assessed using KIDMED score (continuous variable). Sleep duration during weekday (hour/day) was also collected (continuous variable). Mothers' education level was also gathered, categorized as dichotomous (up to high school/university studies). Additionally, information on maternal mental health was collected, including the presence of any diagnosed mental health disorders (no/yes). Finally, socioeconomic status (SES) was collected using a variable of estimated family income based on geolocation (euros/year).

### Statistical analysis

Multivariate linear regression models were computed between the exposure variables (psychological wellbeing, moderate to vigorous physical activity, tobacco and alcohol consumption) and neuropsychological PCs to study their linear associations. Variables involved in research question framework were selected according Directed Acyclic Graph (Supplementary Figure 2). The psychological wellbeing score was treated as a continuous variable as an exposure in regression model. For moderate to vigorous physical activity, we computed the coefficients of linear regression using questionnaire responses as categorical (ranging from “one time or less per week” to “more than three times per week”) and continuous values (1–4). For tobacco and alcohol consumption we used categorical exposure (yes/no), with “never consumption” (no) as the reference value for linear regression The regression models were adjusted for confounding variables, including age, gender and maternal education and SES. Age was treated as a continuous variable (12–16 years old), while sex (female/male) and maternal education (up to high school/university studies) were treated as categorical variables. Finally, SES was treated as a continuous variable (euros/year).

For statistical significance, a *p*-value below 0.05 was considered statistically significant in all regression models. To assess multicollinearity among variables, the Variance Inflation Factor (VIF) was calculated using the datasets employed in the multivariate linear regression models, with VIF values below 5 considered acceptable, and linear model assumptions were visually examined using residual plots, Q–Q plots, residual histograms, and residuals vs. leverage plots. Additionally, we conducted multiple testing correction employing the False Discovery Rate (FDR) test (Benjamini and Hochberg, 1995). As the main criterion, we considered threshold alpha value of 0.10 to compute the *q*-values. Only *p*-values below the *q*-values were considered as new thresholds for statistical significance. After performing the regression models, the population of the WSS main dataset was compared with the population included in the models to assess any selection bias by comparing neuropsychological tests and model confounders (sex, age, maternal education and SES). Sensitivity analyses were performed to assess the robustness of the main model and the confounding effects of relevant variables included in the research question framework. The following variables were examined in the sensitivity analyses: BMI *z*-score, adherence to the MedDiet, sleep duration, maternal mental health, and SES. When SES was analyzed as potential confounding factor in sensitivity analyses, we only adjusted by exposure variable, sex, age and maternal education. The comparisons between the main and sensitivity models were sample-size equalized, and variations of ±10% in the coefficient of exposure variable were considered indicative of a confounding effect. Finally, all analyses were conducted using R base (version 4.4.0) and R Studio (version 4.2.3). The library “zscorer” as used to compute BMI *z*-score (Guevarra, 2019), while “factoextra” and “psych” were employed in PCA (Kassambara and Mundt, 2020; Revelle, 2022).

## Results

The baseline characteristics of the study population sample are shown in Table 1. Participants had an equal gender distribution, and age was within few years of range (mean, 13.85 years, SD = 0.94). Most mothers' participants had university studies (59.0%) and reported no mental health diseases diagnosed (89.0%). Participants had a mean family income (euros/year) of 39528.06 (SD = 10716.73).

**Table 1:** Baseline characteristics of the study population sample.

**Characteristic**	***N***	**Value**
**Neuropsychological function**		
2-back task (*d* prime)^a^	523	3.51 (0.80)
PMA-R (total correct responses)^a^	523	17.16 (5.52)
Roulettes task (CUPRAT)^a^	523	8.10 (4.39)
Inattention—ADHD (number of symptoms)^b^	523	4.34 (5.64)
Hyperactivity—ADHD (number of symptoms)^b^	523	2.30 (4.21)
ERT (total correct responses)^a^	523	48.59 (5.93)
SDQ—externalizing (total score)^b^	523	6.28 (3.28)
**Exposure**		
Psychological wellbeing (KIDSCREEN-27 test, total score)	692	28.20 (4.23)
Physical activity: moderate to vigorous (times/week)	662	
One or less		125 (19%)
Two		167 (25%)
Three		172 (26%)
More than three		198 (30%)
Alcohol consumption^c^	620	
No		374 (60%)
Yes		246 (40%)
Ever gotten drunk	410	
No		315 (77%)
Yes		95 (23%)
Cigarettes consumption^c^	691	
No		578 (84%)
Yes		113 (16%)
**Covariates**		
Sex, *n* (%)	729	
Female		395 (54%)
Male		336 (46%)
Age (years)	729	13.85 (0.94)
Height (m)	724	1.62 (0.09)
Weight (kg)	723	54.33 (11.17)
Body mass index *z*-score	721	0.34 (1.06)
Mediterranean diet adherence (KIDMED score)	641	6.81 (2.14)
Sleep duration—weekday (hour/day)	644	7.99 (0.95)
Maternal education	726	
Non-university		295 (41%)
University		431 (59%)
Maternal mental health disorder	670	
No		599 (89%)
Yes		71 (11%)
Socioeconomic incomes (euros/year)	676	39528.06 (10716.73)

CUPRAT, Roulettes task—total risk adjustment; ERT, Benton Emotional Recognition Task; Hyperactivity-ADHD, Attention Deficit Hyperactivity Disorder test score for Hyperactivity item; Inattention-ADHD, Attention Deficit Hyperactivity Disorder test score for Inattention item; PMA-R, Primary Mental Abilities; SDQ, Strengths and Difficulties Questionnaire.

Data are expressed as mean (standard deviation) in continuous variables and number of sample (%) in categorical variables.

^a^Higher scores indicate better performance.

^b^Higher scores indicate worse performance.

^c^Asked as if they have ever consumed any alcoholic beverage or smoked any normal or electronic cigarette.

The dataset, with the neuropsychological tests, met the assumptions required for PCA. Bartlett's test of sphericity was significant (χ^2^ = 547.33), indicating adequate correlations among variables. In addition, the Kaiser–Meyer–Olkin measure of sampling adequacy was 0.70, confirming that the data were suitable for PC extraction (Supplementary Table 2). PC retention followed the Kaiser criterion (eigenvalue > 1.0; Supplementary Table 3). The eigenvalues were 1.53 for PC1, 1.13 for PC2, and 0.93 for PC3. According to this criterion, only PCs with eigenvalues greater than 1.0 were retained; therefore, PC1 and PC2 were selected. Additionally, the PC1 and PC2 identified accounted for 51.85 % of the total variance of the neuropsychological functions analyzed in the adolescent population sample. The retained PC was also confirmed by the scree plot (Supplementary Figure 3). Based on the scree plot, the percentage of explained variance shows a steep decline from the first to the second component and a more gradual decrease thereafter. The “elbow” appears at PC2, where the curve begins to flatten substantially. This indicates that adding further components yields only marginal increases in explained variance.

**Figure 1:**
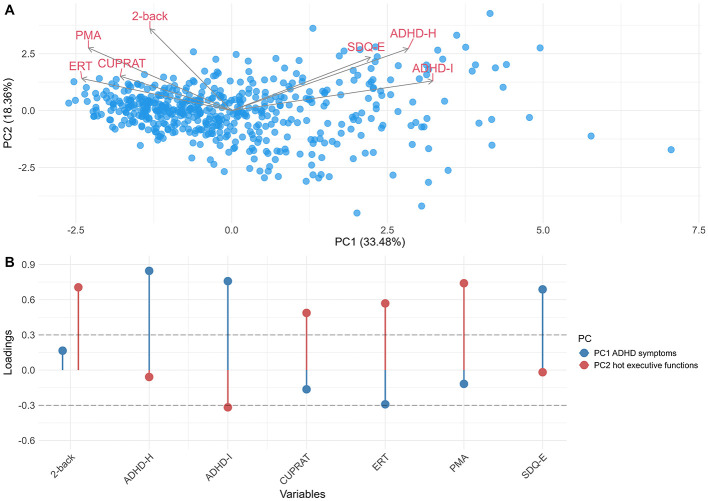
Neuropsychological variables in principal component analysis. Principal component analysis in neuropsychological functions. PC1 represent “ADHD symptoms” and PC2 represent “hot executive functions.” **(A)** Biplot of PC1 and PC2. **(B)** Loadings variable in PC1 and PC2. The two PC retained, before to varimax rotation, add up to 51.85% of the total variance of the neuropsychological functions (cumulative variance). ERT, Emotion Recognition Task; CUPRAT, Roulettes task—total risk adjustment; PMA, Primary Mental Abilities; SDQ-E, Strengths and Difficulties Questionnaire-Externalizing; ADHD-H, Attention Deficit Hyperactivity Disorder test score for Hyperactivity item; ADHD-I, Attention Deficit Hyperactivity Disorder test score for Inattention item; PC, Principal Component.

The factor loadings for each PC are presented in Figure 1 to illustrate the neuropsychological patterns and loadings greater than |0.3| were statistically relevant for explaining variable variance within each PC. The first neuropsychological PC, referred to “ADHD symptoms” PC, was characterized by positive loadings such as ADHD-hyperactivity (loading = 0.84), ADHD inattention (loading = 0.75) and SDQ externalizing problems (loading = 0.68). The second neuropsychological PC, referred to “hot executive functions” PC, was characterized by positive loadings such as PMA-R (loading = 0.74), 2-back (loading = 0.70), ERT (loading = 0.56) and roulettes task (loading = 0.48). ADHD-Inattention score was identified as a negative loading variable (loading = −0.37) in this PC. Standardized scores for the “ADHD symptoms” PC ranged between −1.60 and 4.48 and the standardized scores for the “hot executive functions” PC ranged between −4.12 and 2.01, both scores with a mean of 0. To assess stability of PCs, Pearson correlation was performed along splits 1, 2, and 3. PC1 was highly correlated in split 1, 2, and 3 (Split 1, Split 2 = −0.99; Split 1, Split 2 = −0.98 and Split 2, Split 3 = 0.98). To assess reproducibility of PCs, Bootstrapped PCA was performed (*n* = 1,000). PC1 “ADHD symptoms” showed high load for ADHD inattention and hyperactivity, and PC2 “hot executive functions” for 2-back and PMA-R tasks. However, the confidence interval of the loadings crossed zero (Supplementary Table 4).

Table 2 presents the fully adjusted model associations between the exposures psychological wellbeing and lifestyle behavior (moderate to vigorous physical activity, alcohol consumption, and cigarette use) with the outcomes: the standardized scores for the neuropsychological PCs. We found statistically significant negative associations between psychological wellbeing and the “ADHD symptoms” PC (β_1_ = −0.04, CI = −0.07, −0.02, *p*-value = < 0.001). Additionally, ever having consumed alcohol was positively associated with the “ADHD symptoms” PC (β_1_ = 0.26, 95% CI = 0.07, 0.44, *p*-value = 0.006), compared to the reference group (“never having consumed alcohol”). The results were statistically consistent when participants were asked if they had ever been drunk (“yes”; β_1_ = 0.49, 95% CI = 0.15, 0.83, *p*-value = 0.005) compared with the reference group (“never”). Additionally, a significant and positive association was found between having ever used cigarettes (traditional or electronic) and “ADHD symptoms” PC (β_1_ = 0.66, 95% CI = 0.42, 0.90, *p*-value < 0.001), compared to the reference group (“never having used cigarettes”).

**Table 2:** Multiple linear regression models for psychological wellbeing, lifestyle, and neuropsychological PC in the adolescent population sample.

**Variable**	***n***	**PC1: “ADHD symptoms”**			**PC2: “Hot executive functions”**		
		$\beta_{1}^{\text{a}}$	**95% CI** ^a^	***p*** **-value**	$\beta_{1}^{\text{a}}$	**95% CI** ^a^	***p*** **-value**
Psychological wellbeing (continuous score)	493	−0.04	−0.07, −0.02	**< 0.001**	0.02	−0.01, 0.04	0.164
**Physical activity: moderate to vigorous (times/week)**							
One or less	95	Ref.			Ref.		
Two	132	0.14	−0.11, 0.39	0.277	0.23	−0.03, 0.49	0.080
Three	136	0.15	−0.11, 0.40	0.256	0.33	0.07, 0.59	**0.014**
More than three	154	0.06	−0.19, 0.32	0.620	0.28	0.02, 0.54	**0.033**
Continuous score^b^	517	0.01	−0.07, 0.09	0.759	0.09	0.00, 0.17	**0.041**
**Alcohol consumption**							
Never	279	Ref.			Ref.		
Yes	187	0.26	0.07, 0.44	**0.006**	−0.04	−0.23, 0.14	0.657
**Ever gotten drunk**							
Never	227	Ref.			Ref.		
Yes	50	0.49	0.15, 0.83	**0.005**	0.03	−0.30, 0.36	0.839
**Cigarettes consumption**							
Never	422	Ref.			Ref.		
Yes	75	0.66	0.42, 0.90	**< 0.001**	−0.09	−0.34, 0.16	0.499

PC, Principal Component; n, number of subjects with available data; CI, Confidence Interval; Ref., Reference group.

^a^Beta coefficient (slope) and 95% CI estimated using multiple linear regression models adjusted for sex, age, maternal education, and socioeconomic status. The number of subjects is the same for both neuropsychological principal components.

^b^Physical activity was assessed as self-reported frequency of moderate-to-vigorous activity (times/week) with four ordinal categories: one or less, two, three, and more than three. The “continuous score” represents physical activity treated as a continuous variable derived from the ordinal responses and used as the exposure in the analysis.

Bold values indicate *p*-values less than 0.05.

According to the associations of exposure variables and “hot executive functions” PC, we only found statistically significant and positive associations between “moderate to vigorous physical activity,” as a continuous variable, and “hot executive functions” PC (β_1_ = 0.09, 95% CI = 0.00, 0.17, *p*-value = 0.041). These results were statistically consistent when the exposure was treated as a categorical variable. We found statistical associations for both “three times” (β_1_ = 0.33, 95% CI = 0.07, 0.59, *p*-value = 0.014) and “more than three times” of vigorous physical activity (β_1_ = 0.28, 95% CI = 0.02, 0.54, *p*-value = 0.033) compared to the reference group (“one time or less per week”) in relation to “hot executive functions” PC. All linear regression models showed acceptable multicollinearity levels (VIF < 5.00; Supplementary Figure 4) and met the main assumptions of linear regression, including normality, homoscedasticity, and absence of influential outliers, as evidenced by the Q–Q plots, residual histograms, residual–fitted plots, and leverage diagnostics (Supplementary Figures 5, 6).

Across the models examining psychological wellbeing and physical activity, as well as those assessing alcohol consumption, having ever been drunk, or tobacco use, maternal education consistently showed associations with both neuropsychological PCs, using the same main models and including all covariates (Supplementary Tables 5, 6).

Test significance did not change after correcting *p*-values of the associations between exposure variables and neuropsychological PC for multiple testing using FDR (Supplementary Table 7).

To assess differences for any potential bias in the population with neuropsychological tests, the full dataset and the regression model dataset were compared. No relevant differences were detected (Supplementary Table 8). Additionally, after the sensitivity analyses, we did not observe any confounding effect on the exposures when comparing the main model with the sensitivity models adjusted for BMI *z*-score, adherence to the MedDiet, sleep duration, maternal mental health, and SES (differences between the coefficients were below 10%; Supplementary Tables 9, 10). Only the model of associations between physical activity and “hot executive functions” PC when it was additionally adjusted for BMI *z*-score showed a loss of statistical significance (*p*-value = 0.05) compared with the main model (*p*-value = 0.049), while the relative change in the effect estimate (coefficient) was −0.6%.

## Discussion

In this study, we observed associations between psychological wellbeing, lifestyle behavior and neuropsychological PCs, derived from PCA, among an adolescent population sample. Two neuropsychological PCs were identified with specific characteristics: “ADHD symptoms” and “hot executive functions” PCs. Psychological wellbeing showed a negative association with the “ADHD symptoms” PC, whereas ever having used alcohol or cigarette was positively associated with this PC. Moderate to vigorous physical activity showed a positive association with the “hot executive functions” PC.

Psychological wellbeing was negatively associated with the “ADHD symptoms” PC, which is consistent with the existing literature. Previous research has found that poorer psychological wellbeing is associated with an ADHD diagnosis in children and reduced happiness in their relationships with their families (Peasgood et al., 2016; Bunford et al., 2015). Consistent with our findings, other research revealed a significantly lower association of health-related quality of life in children with ADHD compared to controls. This finding was observed in the KIDSCREEN-52 sub-scales such as individual mood, school environment and social acceptance (López-Villalobos et al., 2019). Similarly, a meta-analysis of 23 studies in the child and adolescent population showed that ADHD was associated with a substantially poorer health-related quality of life for children, including psychological wellbeing (Wanni Arachchige Dona et al., 2023). Psychological wellbeing is linked to cognitive functioning and ADHD symptoms across adolescence and adulthood. It is influenced by factors such as diet quality, parental mental health, and socioeconomic status. No confounding effects of MedDiet adherence, maternal mental health or SES were observed. However, maternal education, included as an adjustment covariate in all models, was inversely associated with the exposures in the “ADHD symptoms” PC models and positively associated with the exposures in the “hot executive functions” PC models. Adolescents from low-income or vulnerable backgrounds often report lower wellbeing, which may relate to poorer cognitive performance or greater ADHD risk (Rakesh et al., 2025). Individuals with ADHD also tend to show difficulties in daily decision-making and a higher likelihood of adverse mental health or social outcomes (Sultan et al., 2021). Even in an adult populations, a study with 11,234 non-institutionalized adults showed that participants with higher psychological wellbeing scored significantly better on a global cognitive measure, after adjusting for depressive symptoms and sociodemographic factors (Llewellyn et al., 2008). From a preventive health perspective, balanced cognitive functioning is a key predictor of Instrumental Activities of Daily Living—such as task planning, transportation, and financial decision-making—in both normal aging and cognitive impairment (Raimo et al., 2024). Taken together, these findings highlight psychological wellbeing as a key factor linked to cognitive functioning, specifically attention functions, and mental health outcomes across the lifespan. Ensuring support for emotional and psychological health from early adolescence may yield long-term benefits for cognitive development, decision-making, and resilience into adulthood.

On the other hand, ever use of alcohol and cigarette use were positively associated with the “ADHD symptoms” PC, meaning that adolescents who ever tried alcohol and/or ever got drunk, or ever smoked cigarettes/e-cigarettes, were more represented in the “ADHD symptoms” PC. Longitudinally, twins who smoked showed a greater increase in attention problems from adolescence to adulthood compared to their never-smoking co-twin (Treur et al., 2015). When ADHD diagnosis is the treated as exposure in the research question, this is in accordance with scientific literature since adolescents with an ADHD diagnose have higher odds of smoking and drinking alcohol (McCabe et al., 2025; Heradstveit et al., 2022). In fact, a study from Rhodes et al. revealed that they are not only more likely to smoke but also tend to start smoking at younger ages and transition to regular smoking more rapidly (Rhodes et al., 2016). Crucially, this relationship may reflect a bidirectional association, rather than a simple cause-effect dynamic. Additionally, certain risk-taking behaviors, such as using alcohol (de Goede et al., 2021) or cannabis, and socializing with peers who use tobacco increase the likelihood of tobacco use (Mahedy et al., 2021). From a neurobiological perspective, the cerebral mechanisms studied in individuals with ADHD involve a core dopamine deficit in the prefrontal cortex and basal ganglia (Wu et al., 2012). This dysregulation affects both tonic and phasic dopamine transmission and is accompanied by alterations in noradrenergic pathways, leading to impairments in executive functions, inhibitory control, and reward processing (Wu et al., 2012). Structural and functional neuroimaging studies consistently report reduced volume and altered activation in regions such as the dorsolateral prefrontal cortex, anterior cingulate cortex, and striatum, supporting the view that ADHD is characterized by a disruption in networks subserving cognitive control and motivational processes (Hart et al., 2013). This neurobiological and brain structural profile is thought to underlie the increased risk for substance use. Specifically, nicotine (from tobacco) triggers a rapid dopamine release, which can momentarily alleviate attentional deficits. Nicotine induces a rapid surge in dopamine secretion within mesolimbic and mesocortical pathways, particularly in the ventral striatum and prefrontal cortex, which can momentarily alleviate attentional deficits and enhance alertness (Morel et al., 2019). By acting on nicotinic acetylcholine receptors located on dopaminergic neurons, nicotine transiently improves arousal, vigilance, and working memory, which may explain why some individuals with ADHD-like symptoms are especially prone to using tobacco as a form of self-medication (Sacco et al., 2004). Similarly, alcohol consumption directly affects the reward system, temporarily reducing both impulsivity and anxiety (Lee et al., 2011) by enhancing GABAergic inhibition and modulating dopaminergic signaling in mesolimbic pathways (Morel et al., 2019).

Conversely, moderate to vigorous physical activity was not significantly associated with the “ADHD symptoms” PC. However, a meta-analysis suggested that physical activity may alleviate ADHD symptoms and improve attention and executive functions (Sun et al., 2022). The characteristics of the WSS participants, particularly that they had symptoms but not enough to meet the criteria for a disease diagnosis, may explain why similar findings were not observed in our research.

Regarding the “hot executive functions” PC, neither tobacco and alcohol use nor psychological wellbeing showed significant associations. Despite this, a higher level of physical activity was a favorable indicator for this PC. Thus, adolescents who performed more vigorous physical activity, outside of the school, had better performance in neuropsychological functions. This neuropsychological PC evaluates fluid intelligence, working memory, and decision-making in risky scenarios. Research suggests that higher levels of aerobic physical activity enhance a range of executive functions, especially working memory and inhibition, in children (Guiney and Machado, 2013). A systematic review of 20 research articles on adolescent population showed that physical activity was positively linked with better academic performance (Esteban-Cornejo et al., 2015). A systematic review and meta-analyses with 35 studies showed that physical activity interventions were associated with significant benefits in overall executive functions and their subdomains. Specifically, improvements were reported in cognitive flexibility, inhibitory control, working memory, and higher-level executive functions (Liu et al., 2025). When the modeling is approached by adjusting executive-functions as exposure; children, adolescents and adults with higher executive function scores tend to be more engaged in physical activity (Gürdere et al., 2023). These findings highlight the complexity of lifestyle habits, which appear to depend bidirectionally on executive functions and may have positive associations with physical activity (reverse causation). The biological mechanisms behind this phenomenon might be explained by the suggestion that aerobic and anaerobic exercise enhance brain development. Thus, both aerobic and anaerobic exercise could promote growth, survival and neural transmission in neurons. Additionally, they increase the brain-derived neurotrophic factor which have been associated with improvements learning and memory (Herting and Chu, 2017). Our findings support the evidence of the positive association between physical activity and hot executive functions in the child and adolescent population sample. It is important to explore this relationship from an adaptive perspective, considering how children and adolescents are learning to move and moving to learn (Guiney and Machado, 2013). That is, while moving they not only improve their physical skills, but also have been linked to social abilities and enhance other skills that may have an impact in their hot executive functions.

Regarding the limitations of the current research, we can identify methodological limitations and measurement limitations. This study is a cross-sectional research, therefore we cannot determine the directionality of the associations due to the nature of the study; therefore causal relationships cannot be inferred. For example, it is unclear whether increased physical activity improves performance in the “hot executive function” PC (Guiney and Machado, 2013), or if individuals with better hot cognition performance might have higher psychosocial factors like self-efficacy and social support, leading to greater adherence to physical activity (Farren et al., 2017). Another limitation is the inevitable residual confounding in epidemiological studies, which prevents us from capturing all possible environmental and non-measured socioeconomic factors that may affect our exposures and neuropsychological outcomes Regarding measurement limitations, the physical activity data collected was not highly reliable, since there was only a question on how many days per week the participant was doing vigorous exercise outside of school. That is, the data was self-reported and limited to one question instead of collecting it with sophisticated devices that might give a more accurate approximation of the participants' level of physical activity. Tobacco use and alcohol consumption were also self-reported, likely leading to underestimation of these behaviors. Also, some participants skipped these questions, which might indicate a higher likelihood of substance use since adolescents often conceal their smoking and drinking habits in self-reported questionnaires (Hwang et al., 2018). Such described measures are susceptible to reporting biases, including under- or overestimation of actual behaviors, which may in turn influence the internal validity of the observed associations. To assess the robustness of our findings in the presence of potential reporting bias, we conducted sensitivity analyses exploring different model specifications and subgroups. While these analyses help evaluate the stability of the observed associations, they do not fully eliminate the inherent limitations of self-reported measures of physical activity, alcohol consumption, and tobacco use. Therefore, results should be interpreted with caution, and future studies incorporating objective assessments are warranted to strengthen the internal validity of the findings. Lastly, regarding the PCA quality control, the PCs demonstrated good stability across the three split samples, particularly PC1 “ADHD symptoms.” However, the confidence intervals obtained from the bootstrap analysis revealed some variability, suggesting that the reproduction of the PCA may not consistently exhibit the same loading pattern across these data. This is because even small variations in the data can lead to changes in the variance in the orthogonal plane, affecting the PCs and their corresponding loadings.

A strength of this study is its comprehensive approach to lifestyle data. For instance, in addition to recording whether participants have ever tried alcohol, we also gathered information on whether they have ever been drunk. Similarly, for smoking we collected data on both traditional cigarettes and e-cigarettes, the latter being a relatively new area of study with emerging evidence on its health implications (Buettner-Schmidt et al., 2024). Another important strength is the use of computerized cognitive tests that specifically measure cognitive functions, rather than relying on more abstract indicators, like school performance scores. We also adopted a novel approach (PCA) to assess the impact of multiple neuropsychological functions of adolescents, which provides better methodological robustness. Moreover, the use of standardized neurocognitive and psychological tests for the PCA brings reliable information about the profile of the adolescent in terms of ADHD symptomatology and executive functions. Finally, the observed associations were robust, remaining consistent across comparisons between the main and sensitivity models, highlighting that our results are unlikely to be substantially influenced by unmeasured confounding.

Overall, our results suggest that lower psychological wellbeing and ever having consumed alcohol and cigarettes may be linked to ADHD-related symptoms, while moderate to vigorous physical activity was positively associated with “hot executive functions” PC in adolescents. These findings are important for understanding neuropsychological functioning related to brain maturity during adolescence. More randomized interventional studies among teenagers are needed to determine the causality of the associations observed in this study. Additionally, these findings suggest important implications for schools and clinical practice. Promoting physical activity through structured physical activity in social spaces or yoga intervention can improve also psychological wellbeing and reduce negative mental health symptoms such anxiety or ADHD symptoms, especially when implemented through school-based programs. Additionally, preventive medical interventions (e.g., population screening and early care) may help facilitate early diagnosis and management of ADHD-like symptom, thereby supporting optimal cognitive development during adolescence. Additionally, future studies incorporating neuroimaging and more diverse populations would further clarify the underlying mechanisms and improve generalizability. However, this study provides valuable insights and a scientific foundation for further investigation into the potential effects of psychological wellbeing and lifestyle on neuropsychological functioning during adolescence.

## Data Availability

The raw data supporting the conclusions of this article will be made available by the authors, without undue reservation.
