# Supplementary material for: Exploring the interplay of neuropsychological functions, psychological wellbeing, and lifestyle through principal component analysis: a comprehensive study
Source: Front Psychol. 2025 Dec 18;16:1692251. doi: 10.3389/fpsyg.2025.1692251 (PMC12756113; doi:10.3389/fpsyg.2025.1692251)
Supplement: Supplementary file 1 [file Supplementary_file_1.docx]

**Supplementary Material**

**Appendix S1. Methods (extended)**

The first primary variable for PCA was n-back task. In the n-back task the adolescent participants were required to monitor a series of stimuli presented in the center of the laptop's screen and they had to respond whenever a given stimulus was the same as the one presented n trials previously (1-, 2-, and 3-back) [1]. Stimuli were displayed for 1500 milliseconds (ms) with a 1000 millisecond inter stimulus period in a constant central place on a white backdrop. Everyone had to push a certain keyboard button when the target appeared on the screen. For the current research we use the 2-back test, where higher scores indicate better working memory performance.

The second primary variable for PC identification was the Test of Primary Mental Abilities (PMA-R) adapted for Spanish population. This test measures the inductive reasoning subtest, fluid intelligence, and analyzing seven abilities: word fluency, verbal comprehension, spatial visualization, number facility, associative memory, reasoning, and perceptual speed [2]. The test used was reasoning, it involved selecting a letter from a list of six options that were beneath a predetermined string of letters. Thus, it addresses letter-based inductive reasoning. The amount of accurate item responses determines the final score. A higher score also denotes greater fluid intelligence proficiency.

The third primary variable to perform PC was the Cups Task Risk adjustment Total score (CUPRAT) from the Roulettes Task. This test was a gambling experiment designed to evaluate risky decision-making, specifically whether individuals modify their risky behaviors following the probabilities and significance of the outcome (cognition affected by emotion) [3]. The scores are measured as total risk adjustment and the closer to 0 the risk adjustment index is, the more risk insensitivity.

The fourth primary variable to identify PC was the Benton Emotional Recognition Task (ERT). The psychometric test is a computer-generated image for assessing the recognition of six fundamental facial emotional expressions: anger, contempt, fear, pleasure, sadness, and surprise. One at a time, the screen shows computer-generated pictures that have been warped from actual people's facial characteristics to represent several emotions [4]. Each image is shown for 200 ms and then immediately covered up to prevent residual processing of the image. Finally, based on the facial expression, the participant must select one of six possible emotions. A total of 60 images were used for emotion recognition. The measures for ERT cover the correct total responses of facial emotions, thus higher scores indicate better emotional recognition.

The fifth and sixth primary variables to identify PC were the inattention and hyperactivity scores obtained from an ADHD-symptom checklist of “The Diagnostic and Statistical Manual of Mental Disorders- IV (American Psychiatric Association)” [5]. First, the inattention score was calculated adding the results of item 1 to item 9 and hyperactivity score was computed adding the results of item 10 to item 18. These scores were used only as dimensional variables to reflect number of symptoms, without applying any cut-off points for an ADHD diagnosis. For both variables, high values scores indicate more ADHD symptoms.

The seventh primary variable was the Strengths and Difficulties Questionnaire (SDQ), a child mental health assessment tool with five hypothesized subscales. The scale was originally developed for the measurement of five aspects related to mental health screening namely four “difficulty” domains: hyperactivity/inattention, emotional symptoms, conduct problems, and peer problems. The SDQ additionally gathers data on prosocial behavior as a strength domain [6]. The SDQ externalizing score, which ranges from 0 to 20, is calculated by adding the conduct problems and hyperactivity scales, and a higher score indicates abnormal behaviors.

**References**

[1] López-Vicente M, Forns J, Suades-González E, et al. Developmental Trajectories in Primary Schoolchildren Using n-Back Task. Front Psychol 2016;7.

[2] THURSTONE TG. The Tests of Primary Mental Abilities. Pers Guid J 1957;35:569–76.

[3] Levin IP, Weller JA, Pederson A, et al. Age-related differences in adaptive decision making: Sensitivity to expected value in risky choice. Judgm Decis Mak 2007.

[4] Montagne B, Kessels RPC, De Haan EHF, et al. The Emotion Recognition Task: A Paradigm to Measure the Perception of Facial Emotional Expressions at Different Intensities. Percept Mot Skills 2007;104:589–98.

[5] American Psychiatric Association. Diagnostic and Statistical Manual of Mental Disorders: DSMIV-TR®. Washington: 2000.

[6] Goodman R. The Strengths and Difficulties Questionnaire: A Research Note. J Child Psychol Psychiatry 1997;38:581–6.

Supplementary Table 1. Internal consistency of neuropsychological tests.

| Neuropsychological test | Cronbach’s alpha | 95% CI |
| --- | --- | --- |
| ADHD – inattention | 0.96 | 0.96 , 0.96 |
| ADHD – hyperactivity | 0.92 | 0.93, 0.94 |
| SDQ – Conduct problems  (items 5, 7, 12, 18, 22) | 0.53 | 0.48, 0.57 |
| SDQ - Hyperactivity/Inattention (items 2, 10, 15, 21, 25) | 0.71 | 0.68, 0.74 |

Hyperactivity-ADHD Attention Deficit Hyperactivity disorder test score for hyperactivity item, Inattention-ADHD Attention Deficit Hyperactivity Disorder test score for inattention item, SDQ Strengths and Difficulties Questionnaire externalizing symptoms.

Supplementary Table 2. Bartlett’s test of sphericity and Kaiser-Meyer-Olkin factor adequacy in neuropsychological tests.

| **Test** | | **Value** | **Interpretation** |
| --- | --- | --- | --- |
| Bartlett’s test of sphericity | Chi-square | 547.33^a^ | The data is suitable for PCA |
| Kaiser-Meyer-Olkin factor adequacy | Overall MSA | 0.70 | Data is suitable to perform PCA |

PCA principal component analysis.

^a^ p-value <0.001.

Supplementary Table 3**.** Components and eigenvalues in principal components analysis.

| **Component** | **Eigenvalues** | **Variance (%)** | **Cumulative Variance (%)** |
| --- | --- | --- | --- |
| 1 ^a^ | 1.53^a^ | 33.48 | 33.48 |
| 2 ^a^ | 1.13^a^ | 18.36 | 51.85 |
| 3 | 0.93 | 12.44 | 64.29 |
| 4 | 0.88 | 11.20 | 75.49 |
| 5 | 0.84 | 10.21 | 85.70 |
| 6 | 0.78 | 8.82 | 94.52 |
| 7 | 0.61 | 5.47 | 100 |

^a^ Components selected for principal component analysis according Kaiser criteria (eigenvalues >1.00).

Supplementary table 4. Loadings of principal components.

| PC | Variable | Loading | Varimax rotation | Bootstrapped loadings  Mean (95% CI) ^a^ | Split  1 ^b^ | Split  2 ^b^ | Split  3 ^b^ | Pearson ^b^ |
| --- | --- | --- | --- | --- | --- | --- | --- | --- |
| PC 1 | 2 back (d’) | -0.32 | 0.16 | -0.16 (-0.37, 0.35) | 0.27 | -0.24 | -0.07 | PC1 S1, S2  -0.99 |
|  | PMA | -0.56 | -0.11 | -0.29 (-0.60, 0.59) | 0.39 | -0.37 | -0.29 |  |
|  | CUPRAT | -0.43 | -0.16 | -0.23 (-0.45, 0.45) | 0.27 | -0.28 | -0.29 | PC1 S1, S3  -0.98 |
|  | ADHD-Hyperactivity | 0.69 | 0.84 | 0.37 (-0.72, 0.74) | -0.42 | 0.43 | 0.50 |  |
|  | ADHD-Inattention | 0.78 | 0.75 | 0.42 (-0.80, 0.80) | -0.48 | 0.48 | 0.57 | PC1 S2, S3  0.98 |
|  | SDQ - Externalizing | 0.54 | 0.68 | 0.29 (-0.56, 0.58**)** | -0.34 | 0.40 | 0.33 |  |
|  | ERT | -0.58 | -0.29 | -0.31 (-0.60, 0.60) | 0.40 | -0.36 | -0.36 |  |
| PC 2 | 2 back (d’) | 0.65 | 0.70 | -0.05 (-0.68, 0.67) | 0.54 | -0.54 | 0.55 | PC2 S1, S2  -0.17 |
|  | PMA | 0.49 | 0.74 | -0.04 (-0.55, 0.54) | 0.40 | -0.42 | 0.48 |  |
|  | CUPRAT | 0.27 | 0.48 | -0.02 (0.31, 0.31) | 0.40 | -0.08 | 0.24 | PC2 S1, S3  0.39 |
|  | ADHD -Hyperactivity | 0.49 | -0.05 | -0.04 (-0.54, 0.54) | 0.49 | -0.38 | 0.34 |  |
|  | ADHD-Inattention | 0.23 | -0.31 | -0.02 (-0.29 – 0.29) | 0.27 | -0.24 | 0.09 | PC2 S2, S3  -0.79 |
|  | SDQ - Externalizing | 0.42 | -0.01 | -0.02 (-0.31, 0.30) | 0.23 | -0.40 | 0.41 |  |
|  | ERT | 0.25 | 0.56 | -0.02 (-0.46, 0.46) | 0.03 | -0.37 | 0.30 |  |

PC principal component, CUPRAT Roulettes task – total risk adjustment, ERT Benton Emotional Recognition Task, Hyperactivity-ADHD Attention Deficit Hyperactivity disorder test score for hyperactivity item, Inattention-ADHD Attention Deficit Hyperactivity Disorder test score for inattention item, PMA-R Primary Mental abilities, SDQ Strengths and Difficulties Questionnaire.

^a^ Bootstrapping of principal component analyses using boot function. The bootstrapping was applied on raw loadings to obtain mean and 95%CI (Number of iterations = 1000).

^b^ Random splits of the full sample were performed for PCA, using one-third of the data in each split (total n = 523). Raw loadings of each neuropsychological variable were extracted for each split. Additionally, Pearson correlations were computed to assess the stability of the principal components between split 1, 2 and 3.

**Supplementary Table 5.** Multiple linear regression models for psychological wellbeing, physical activity, and neuropsychological PC in the adolescent population sample.

| **Characteristic** | **PC1 “ADHD symptoms”** | | | | **PC2 “Hot executive functions”** | | | |
| --- | --- | --- | --- | --- | --- | --- | --- | --- |
|  | **n** | $\boldsymbol{\beta}_{\boldsymbol{1}}$**^a^** | **95% CI ^a^** | **p-value** | **n** | $\boldsymbol{\beta}_{\boldsymbol{1}}$ **^a^** | **95% CI ^a^** | **p-value** |
| **Main model: Psychological well-being** | | | | | | | | |
| Psychological well-being | 493 | -0.04 | -0.07, -0.02 | **<0.001** | 493 | 0.02 | -0.01, 0.04 | 0.164 |
| Maternal education |  |  |  |  |  |  |  |  |
| Up to secondary | 202 | Ref. |  |  | 202 | Ref. |  |  |
| University | 291 | -0.30 | -0.47, -0.12 | **0.001** | 291 | 0.29 | 0.11, 0.48 | **0.002** |
| Sex |  |  |  |  |  |  |  |  |
| Female | 280 | Ref. |  |  | 280 | Ref. |  |  |
| Male | 213 | 0.62 | 0.45, 0.80 | **<0.001** | 213 | -0.09 | -0.27, 0.09 | 0.314 |
| Age | 493 | 0.00 | -0.10, 0.09 | 0.920 | 493 | 0.13 | 0.04, 0.23 | **0.006** |
| SES | 493 | 0.00 | 0.00, 0.00 | 0.202 | 493 | 0.00 | 0.00, 0.00 | 0.501 |
| **Main model: Physical activity (categorical value)** | | | | | | | | |
| Physical activity |  |  |  |  |  |  |  |  |
| One or less | 95 | Ref. |  |  | 95 | Ref. |  |  |
| Two | 132 | 0.14 | -0.11, 0.39 | 0.277 | 132 | 0.23 | -0.03, 0.49 | 0.080 |
| Three | 136 | 0.15 | -0.11, 0.40 | 0.256 | 136 | 0.33 | 0.07, 0.59 | **0.014** |
| More than three | 154 | 0.06 | -0.19, 0.32 | 0.620 | 154 | 0.28 | 0.02, 0.54 | **0.033** |
| Maternal education |  |  |  |  |  |  |  |  |
| Up to secondary | 209 | Ref. |  |  | 209 | Ref. |  |  |
| University | 308 | -0.23 | -0.40, -0.06 | **0.010** | 308 | 0.27 | 0.09, 0.45 | **0.003** |
| Sex |  |  |  |  |  |  |  |  |
| Female | 289 | Ref. |  |  | 289 | Ref. |  |  |
| Male | 228 | 0.50 | 0.33, 0.67 | **<0.001** | 228 | -0.17 | -0.35, 0.01 | 0.066 |
| Age | 517 | 0.02 | -0.07, 0.11 | 0.722 | 517 | 0.12 | 0.03, 0.21 | **0.012** |
| SES | 517 | 0.00 | 0.00, 0.00 | 0.187 | 517 | 0.00 | 0.00, 0.00 | 0.201 |
| **Main model: Physical activity (continuous value)** | | | | | | | | |
| Physical activity | 517 | 0.01 | -0.07, 0.09 | 0.759 | 517 | 0.09 | 0.00, 0.17 | **0.041** |
| Maternal education |  |  |  |  |  |  |  |  |
| Up to secondary | 209 | Ref. |  |  | 209 | Ref. |  |  |
| University | 308 | -0.23 | -0.40, -0.06 | **0.009** | 308 | 0.27 | 0.09, 0.45 | **0.003** |
| Sex |  |  |  |  |  |  |  |  |
| Female | 289 | Ref. |  |  | 289 | Ref. |  |  |
| Male | 228 | 0.49 | 0.32, 0.67 | **<0.001** | 228 | -0.17 | -0.35, 0.00 | 0.056 |
| Age | 517 | 0.02 | -0.07, 0.11 | 0.681 | 517 | 0.12 | 0.03, 0.21 | **0.009** |
| SES | 517 | 0.00 | 0.00, 0.00 | 0.183 | 517 | 0.00 | 0.00, 0.00 | 0.208 |

PC principal component, Ref reference group, SES socioeconomic status.

^a^ Beta coefficient (slope) and 95% CI estimated using multiple linear regression models adjusted for sex, age, maternal education and socioeconomic status.

**Supplementary Table 6.** Multiple linear regression models for alcohol, tobacco consumption, and neuropsychological PC in the adolescent population sample.

| **Characteristic** | **PC1 “ADHD symptoms”** | | | | **PC2 “Hot executive functions”** | | | |
| --- | --- | --- | --- | --- | --- | --- | --- | --- |
|  | **n** | $\boldsymbol{\beta}_{\boldsymbol{1}}$**^a^** | **95% CI ^a^** | **p-value** | **n** | $\boldsymbol{\beta}_{\boldsymbol{1}}$ **^a^** | **95% CI ^a^** | **p-value** |
| **Main model: Alcohol consumption** | | | | | | | | |
| Alcohol consumption |  |  |  |  |  |  |  |  |
| Never | 279 |  |  |  | 279 | Ref. |  |  |
| Yes | 187 | 0.26 | 0.07, 0.44 | **0.006** | 187 | -0.04 | -0.23, 0.14 | 0.657 |
| Maternal education |  |  |  |  |  |  |  |  |
| Up to secondary | 187 | Ref. |  |  | 187 | Ref. |  |  |
| University | 279 | -0.23 | -0.41, -0.05 | **0.011** | 279 | 0.27 | 0.09, 0.46 | **0.004** |
| Sex |  |  |  |  |  |  |  |  |
| Female | 260 | Ref. |  |  | 260 | Ref. |  |  |
| Male | 206 | 0.51 | 0.34, 0.68 | **<0.001** | 206 | -0.13 | -0.30, 0.05 | 0.162 |
| Age | 466 | -0.01 | -0.11, 0.09 | 0.813 | 466 | 0.11 | 0.01, 0.21 | **0.035** |
| SES | 466 | 0.00 | 0.00, 0.00 | 0.499 | 466 | 0.00 | 0.00, 0.00 | **0.021** |
| **Main model: Ever gotten drunk** | | | | | | | | |
| Ever gotten drunk |  |  |  |  |  |  |  |  |
| Never | 227 | Ref. |  |  | 227 | Ref. |  |  |
| Yes | 50 | 0.49 | 0.15, 0.83 | **0.005** | 50 | 0.03 | -0.30, 0.36 | 0.839 |
| Maternal education |  |  |  |  |  |  |  |  |
| Up to secondary | 119 | Ref. |  |  | 119 | Ref. |  |  |
| University | 158 | -0.10 | -0.35, 0.15 | 0.420 | 158 | 0.18 | -0.06, 0.41 | 0.150 |
| Sex |  |  |  |  |  |  |  |  |
| Female | 157 | Ref. |  |  | 157 | Ref. |  |  |
| Male | 120 | 0.54 | 0.29, 0.78 | **<0.001** | 120 | -0.14 | -0.38, 0.10 | 0.240 |
| Age | 277 | -0.09 | -0.22, 0.05 | 0.216 | 277 | 0.17 | 0.04, 0.30 | **0.011** |
| SES | 277 | 0.00 | 0.00, 0.00 | **0.024** | 277 | 0.00 | 0.00, 0.00 | 0.899 |
| **Main Model: Cigarettes consumption** | | | | | | | | |
| Cigarettes consumption |  |  |  |  |  |  |  |  |
| Never | 422 | Ref. |  |  | 422 | Ref. |  |  |
| Yes | 75 | 0.66 | 0.42, 0.90 | **<0.001** | 75 | -0.09 | -0.34, 0.16 | 0.499 |
| Maternal education |  |  |  |  |  |  |  |  |
| Up to secondary | 202 | Ref. |  |  | 202 | Ref. |  |  |
| University | 295 | -0.23 | -0.40, -0.05 | **0.012** | 295 | 0.28 | 0.10, 0.46 | **0.003** |
| Sex |  |  |  |  |  |  |  |  |
| Female | 278 | Ref. |  |  | 278 | Ref. |  |  |
| Male | 219 | 0.55 | 0.39, 0.72 | **<0.001** | 219 | -0.12 | -0.29, 0.06 | 0.184 |
| Age | 497 | -0.06 | -0.16, 0.03 | 0.194 | 497 | 0.12 | 0.02, 0.22 | **0.014** |
| SES | 497 | 0.00 | 0.00, 0.00 | 0.224 | 497 | 0.00 | 0.00, 0.00 | 0.183 |

PC principal component, Ref reference group, SES socioeconomic status.

^a^ Beta coefficient (slope) and 95% CI estimated using multiple linear regression models adjusted for sex, age, maternal education and socioeconomic status.

Supplementary Table 7**.** Multivariate regression models (Table 2) corrected p-values for multiple testing using the Benjamini-Hochberg false discovery rate.

| **Exposure** | **Outcome** | **p-value ^a^** | **rank** | **q-value** | **Accepted after FDR correction** |
| --- | --- | --- | --- | --- | --- |
| psychological well-being | PC1 | <0.001 | 1 | 0.010 | Yes |
| cigarettes consumption | PC1 | <0.001 | 2 | 0.020 | Yes |
| ever gotten drunk | PC1 | 0.005 | 3 | 0.030 | Yes |
| alcohol consumption | PC1 | 0.006 | 4 | 0.040 | Yes |
| physical activity | PC2 | 0.041 | 5 | 0.050 | Yes |
| psychological well-being | PC2 | 0.164 | 6 | 0.060 | No |
| cigarettes consumption | PC2 | 0.499 | 7 | 0.070 | No |
| alcohol consumption | PC2 | 0.657 | 8 | 0.080 | No |
| physical activity | PC1 | 0.759 | 9 | 0.090 | No |
| ever gotten drunk | PC2 | 0.839 | 10 | 0.100 | No |

PC1= “ADHD symptoms”, PC2= “Hot executive functions”.

^a^ p-value for continuous exposure variable.

False Discovery Rate (FDR) significance threshold was defined at 0.10. After assessing multivariate linear regression, only the p-values corresponding to the coefficients of the exposure variable are used in FDR comparisons. Coefficients are considered statistically significant if their p-values are smaller than their q-values.

Supplementary Table 8. Comparison of variable information: WSS maximum dataset vs. linear regression model dataset

| Variable | n | Full WSS dataset ^a^ | n | Model dataset ^b^ |
| --- | --- | --- | --- | --- |
| 2 back (d’) | 605 | 3.51 (0.77) | 523 | 3.51 (0.80) |
| PMA | 609 | 16.94 (5.49) | 523 | 17.16 (5.52) |
| CUPRAT | 608 | 8.23 (4.28) | 523 | 8.10 (4.39) |
| ADHD-Hyperactivity | 554 | 2.21 (3.98) | 523 | 2.30 (4.21) |
| ADHD-Inattention | 561 | 4.68 (6.01) | 523 | 4.34 (5.64) |
| ERT | 608 | 48.69 (5.79) | 523 | 48.59 (5.93) |
| SDQ - Externalizing | 571 | 6.25 (3.29) | 523 | 6.28 (3.28) |
| Sex | 729 |  | 493 |  |
| Female |  | 395 (54%) |  | 280 (57%) |
| Male |  | 336 (46%) |  | 213 (43%) |
| Age | 729 | 13.85 (0.94) | 493 | 13.80 (0.92) |
| Maternal education | 726 |  | 493 |  |
| Non-university |  | 295 (41%) |  | 204 (41%) |
| University |  | 431 (59%) |  | 292 (59%) |
| Socioeconomic status | 676 | 39528.06 (10716.73) | 493 | 39228.29 (10166.23) |

WSS Walnuts Smart Snack Intervention Trial, CUPRAT Roulettes task – total risk adjustment, ERT Benton Emotional Recognition Task, Hyperactivity-ADHD Attention Deficit Hyperactivity disorder test score for hyperactivity item, Inattention-ADHD Attention Deficit Hyperactivity Disorder test score for inattention item, PMA-R Primary Mental abilities, SDQ Strengths and Difficulties Questionnaire.

Data are expressed as mean (standard deviation) in continuous variables and number of sample (%) in categorical variables.

^a^ Maximum data of adolescents that participate in WSS.

^b^ Maximum data of adolescents with completed neuropsychological test to compute principal component analyses. Sociodemographic information such as sex, age and maternal education was obtained from dataset of linear regression model between neuropsychological wellbeing and principal component 1 “ADHD symptoms”.

| **Sensitivity variable** | **Characteristics** | **Main model ^a^** | | | | **Sensitivity model ^b^** | | |  |
| --- | --- | --- | --- | --- | --- | --- | --- | --- | --- |
|  |  | **n** | $\boldsymbol{\beta}_{\boldsymbol{1}}$ | **95% CI** | **p-value** | $\boldsymbol{\beta}_{\boldsymbol{1}}$ | **95% CI** | **p-value** | **Δ%** $\boldsymbol{\beta}_{\boldsymbol{1}}$ |
| BMI | PW | 490 | -0.045 | -0.066, -0.023 | **<0.001** | -0.046 | -0.067, -0.024 | **<0.001** | -1.6 |
| MedDiet | PW | 487 | -0.047 | -0.068, -0.026 | **<0.001** | -0.047 | -0.068, -0.026 | **<0.001** | -1.6 |
| Sleep | PW | 486 | -0.042 | -0.063, -0.020 | **<0.001** | -0.042 | -0.064, -0.021 | **<0.001** | -0.8 |
| MMH | PW | 488 | -0.044 | -0.065, -0.022 | **<0.001** | -0.043 | -0.065, -0.022 | **<0.001** | 0.6 |
| SES | PW | 493 | -0.045 | -0.066, -0.024 | **<0.001** | -0.044 | -0.066, -0.023 | **<0.001** | 1.4 |
| BMI | PA | 516 | 0.015 | -0.064, 0.094 | 0.712 | 0.015 | -0.065, 0.094 | 0.713 | -0.3 |
| MedDiet | PA | 511 | 0.018 | -0.060, 0.096 | 0.644 | 0.018 | -0.061, 0.096 | 0.660 | -4.4 |
| Sleep | PA | 513 | 0.009 | -0.070, 0.088 | 0.823 | 0.009 | -0.070, 0.089 | 0.816 | 4.4 |
| MMH | PA | 512 | 0.019 | -0.060, 0.098 | 0.639 | 0.021 | -0.058, 0.101 | 0.601 | 11.9 |
| SES | PA | 517 | 0.015 | -0.064, 0.094 | 0.711 | 0.012 | -0.067, 0.092 | 0.759 | -17.0 |
| BMI | alcohol    no | 277 | Ref |  |  | Ref |  |  |  |
|  | yes | 186 | 0.260 | 0.077, 0.442 | **0.005** | 0.260 | 0.077, 0.443 | **0.005** | 0.2 |
| MedDiet | alcohol    no | 277 | Ref |  |  | Ref |  |  |  |
|  | yes | 184 | 0.279 | 0.097, 0.460 | **0.003** | 0.280 | 0.098, 0.461 | **0.003** | 0.4 |
| Sleep | alcohol    no | 276 | Ref |  |  | Ref |  |  |  |
|  | yes | 184 | 0.266 | 0.083, 0.448 | **0.005** | 0.265 | 0.081, 0.448 | **0.005** | -0.4 |
| MMH | alcohol    no | 277 | Ref |  |  | Ref |  |  |  |
|  | yes | 185 | 0.257 | 0.077, 0.437 | **0.005** | 0.259 | 0.079, 0.439 | **0.005** | 0.8 |
| SES | alcohol    no | 279 | Ref |  |  | Ref |  |  |  |
|  | Yes | 187 | 0.256 | 0.075, 0.438 | **0.006** | 0.255 | 0.074, 0.437 | **0.006** | -0.3 |
| BMI | drunk ever    no | 226 | Ref |  |  | Ref |  |  |  |
|  | yes | 50 | 0.489 | 0.146, 0.832 | **0.005** | 0.489 | 0.146, 0.833 | **0.005** | 0.1 |
| MedDiet | drunk ever     no | 223 | Ref |  |  | Ref |  |  |  |
|  | yes | 49 | 0.506 | 0.172, 0.841 | **0.003** | 0.502 | 0.167, 0.837 | **0.003** | -0.9 |
| Sleep | drunk ever     no | 223 | Ref |  |  | Ref |  |  |  |
|  | yes | 50 | 0.490 | 0.148, 0.831 | **0.005** | 0.476 | 0.133, 0.819 | **0.007** | -2.8 |
| MMH | drunk ever     no | 225 | Ref |  |  | Ref |  |  |  |
|  | yes | 49 | 0.451 | 0.109, 0.792 | **0.010** | 0.455 | 0.113, 0.797 | **0.009** | 1.0 |
| SES | drunk ever     no | 227 | Ref |  |  | Ref |  |  |  |
|  | yes | 50 | 0.468 | 0.124, 0.812 | **0.008** | 0.489 | 0.147, 0.830 | **0.005** | 4.4 |
| BMI | cigarette    no | 419 | Ref |  |  | Ref |  |  |  |
|  | yes | 75 | 0.661 | 0.418, 0.904 | **<0.001** | 0.661 | 0.418, 0.905 | **<0.001** | 0.1 |
| MedDiet | cigarette    no | 416 | Ref |  |  | Ref |  |  |  |
|  | yes | 74 | 0.683 | 0.446, 0.921 | **<0.001** | 0.685 | 0.447, 0.922 | **<0.001** | 0.2 |
| Sleep | cigarette    no | 415 | Ref |  |  | Ref |  |  | - |
|  | yes | 75 | 0.662 | 0.420, 0.904 | **<0.001** | 0.660 | 0.418, 0.903 | **<0.001** | -0.2 |
| MMH | cigarette    no | 419 | Ref |  |  | Ref |  |  |  |
|  | yes | 74 | 0.627 | 0.386, 0.868 | **<0.001** | 0.627 | 0.386, 0.869 | **<0.001** | 0.0 |
| SES | cigarette    no | 422 | Ref |  |  | Ref |  |  |  |
|  | yes | 75 | 0.659 | 0.417, 0.902 | **<0.001** | 0.657 | 0.415, 0.899 | **<0.001** | -0.3 |

**Supplementary Table 9.** Sensitivity analyses. Multiple linear regression models between psychological wellbeing, physical activity, alcohol and cigarette consumption with “ADHD symptoms” principal component.

PW psychological well-being, PA physical activity, BMI body mass index, MedDiet Mediterranean Diet, MMH maternal mental health, SES socioeconomic status, CI confidence interval, Ref reference group.

^a^ Beta coefficient (slope) and 95% CI estimated using multiple linear regression models adjusted for sex, age, maternal education and SES.

^b^ Beta coefficient (slope) and 95% CI estimated using multiple linear regression models adjusted for sex, age, maternal education, SES and the variable of sensitivity analyses. The additional adjustment included BMI z-score or KIDMED score or sleep duration or maternal mental health. When SES was assessed through the sensitivity model, the mail model was only adjusted by sex, age, and maternal education. For model comparisons, dataset samples size were equalized. Differences between the beta coefficient of main model and the sensitivity model are also shown relative to each other (Δ% $\beta_{1}$).

| **Sensitivity variable** | **Characteristics** | **Main model ^a^** | | | | **Sensitivity model ^b^** | | |  |
| --- | --- | --- | --- | --- | --- | --- | --- | --- | --- |
|  |  | **n** | $\boldsymbol{\beta}_{\boldsymbol{1}}$ | **95% CI** | **p-value** | $\boldsymbol{\beta}_{\boldsymbol{1}}$ | **95% CI** | **p-value** | **Δ%**  $\boldsymbol{\beta}_{\boldsymbol{1}}$ |
| BMI | PW | 490 | 0.015 | -0.007, 0.037 | 0.181 | 0.013 | -0.009, 0.036 | 0.244 | -11.5 |
| MedDiet | PW | 487 | 0.015 | -0.007, 0.037 | 0.185 | 0.013 | -0.009, 0.035 | 0.244 | -11.5 |
| Sleep | PW | 486 | 0.018 | -0.004, 0.040 | 0.110 | 0.020 | -0.002, 0.042 | 0.072 | 13.3 |
| MMH | PW | 488 | 0.016 | -0.006, 0.037 | 0.161 | 0.017 | -0.005, 0.039 | 0.129 | 9.0 |
| SES | PW | 493 | 0.016 | -0.006, 0.038 | 0.154 | 0.016 | -0.006, 0.037 | 0.164 | -2.1 |
| BMI | PA | 516 | 0.082 | 0.000, 0.164 | **0.049** | 0.081 | 0.000, 0.163 | 0.050 | -0.6 |
| MedDiet | PA | 511 | 0.076 | -0.006, 0.158 | 0.068 | 0.072 | -0.010, 0.153 | 0.087 | -5.9 |
| Sleep | PA | 513 | 0.075 | -0.007, 0.157 | 0.072 | 0.079 | -0.003, 0.161 | 0.058 | 5.2 |
| MMH | PA | 512 | 0.091 | 0.009, 0.173 | **0.030** | 0.094 | 0.011, 0.176 | **0.026** | 2.9 |
| SES | PA | 517 | 0.083 | 0.001, 0.164 | **0.047** | 0.085 | 0.004, 0.167 | **0.041** | 3.0 |
| BMI | alcohol    no | 277 | Ref |  |  | Ref |  |  |  |
|  | yes | 186 | -0.044 | -0.232, 0.145 | 0.649 | -0.045 | -0.234, 0.143 | 0.638 | -3.4 |
| MedDiet | alcohol    no | 277 | Ref |  |  | Ref |  |  |  |
|  | yes | 184 | -0.055 | -0.243, 0.134 | 0.570 | -0.053 | -0.241, 0.135 | 0.581 | 2.9 |
| Sleep | alcohol    no | 276 | Ref |  |  | Ref |  |  |  |
|  | yes | 184 | -0.035 | -0.223, 0.152 | 0.712 | -0.046 | -0.234, 0.142 | 0.631 | -30.3 |
| MMH | alcohol    no | 277 | Ref |  |  | Ref |  |  |  |
|  | yes | 185 | -0.042 | -0.229, 0.145 | 0.657 | -0.044 | -0.231, 0.143 | 0.643 | -4.6 |
| SES | alcohol    no | 279 | Ref |  |  | Ref |  |  |  |
|  | yes | 187 | -0.046 | -0.234, 0.143 | 0.635 | -0.042 | -0.230, 0.145 | 0.657 | 6.8 |
| BMI | drunk ever    no | 226 | Ref |  |  | Ref |  |  |  |
|  | yes | 50 | 0.040 | -0.291, 0.370 | 0.814 | 0.038 | -0.293, 0.368 | 0.823 | -5.2 |
| MedDiet | drunk ever     no | 223 | Ref |  |  | Ref |  |  |  |
|  | yes | 49 | 0.040 | -0.292, 0.372 | 0.814 | 0.048 | -0.284, 0.381 | 0.775 | 21.9 |
| Sleep | drunk ever     no | 223 | Ref |  |  | Ref |  |  |  |
|  | yes | 50 | 0.049 | -0.280, 0.379 | 0.768 | 0.026 | -0.305, 0.357 | 0.877 | -47.2 |
| MMH | drunk ever     no | 225 | Ref |  |  | Ref |  |  |  |
|  | yes | 49 | 0.021 | -0.311, 0.353 | 0.900 | 0.028 | -0.304, 0.360 | 0.869 | 31.8 |
| SES | drunk ever     no | 227 | Ref |  |  | Ref |  |  |  |
|  | yes | 50 | 0.033 | -0.296, 0.362 | 0.843 | 0.034 | -0.296, 0.364 | 0.839 | 3.3 |
| BMI | cigarette   no | 419 | Ref |  |  | Ref |  |  |  |
|  | yes | 75 | -0.082 | -0.332, 0.168 | 0.521 | -0.079 | -0.329, 0.172 | 0.538 | 4.2 |
| MedDiet | cigarette    no | 416 | Ref |  |  | Ref |  |  |  |
|  | yes | 74 | -0.084 | -0.334, 0.166 | 0.511 | -0.080 | -0.330, 0.170 | 0.530 | 4.5 |
| Sleep | cigarette    no | 415 | Ref |  |  | Ref |  |  |  |
|  | yes | 75 | -0.086 | -0.335, 0.163 | 0.498 | -0.092 | -0.341, 0.157 | 0.468 | -7.1 |
| MMH | cigarette    no | 419 | Ref |  |  | Ref |  |  |  |
|  | yes | 74 | -0.103 | -0.352, 0.146 | 0.417 | -0.103 | -0.353, 0.146 | 0.417 | 0.0 |
| SES | cigarette    no | 422 | Ref |  |  | Ref |  |  |  |
|  | yes | 75 | -0.088 | -0.338, 0.161 | 0.487 | -0.086 | -0.336, 0.164 | 0.499 | 2.8 |

**Supplementary Table 10.** Sensitivity analyses. Multiple linear regression models between psychological wellbeing, physical activity, alcohol and cigarette consumption with “hot executive functions” principal component.

PW psychological well-being, PA physical activity, BMI body mass index, MedDiet Mediterranean Diet, MMH maternal mental health, SES socioeconomic status, CI confidence interval, Ref reference group.

^a^ Beta coefficient (slope) and 95% CI estimated using multiple linear regression models adjusted for sex, age, maternal education and SES.

^b^ Beta coefficient (slope) and 95% CI estimated using multiple linear regression models adjusted for sex, age, maternal education, SES and the variable of sensitivity analyses. The additional adjustment included BMI z-score or KIDMED score or sleep duration or maternal mental health. When SES was assessed through the sensitivity model, the mail model was only adjusted by sex, age, and maternal education. For model comparisons, dataset samples size were equalized. Differences between the beta coefficient of main model and the sensitivity model are also shown relative to each other (Δ% $\beta_{1}$).

**Supplementary Figure 1**. Flowchart of “Walnuts Smart Snack Intervention Trial” participants of the study.


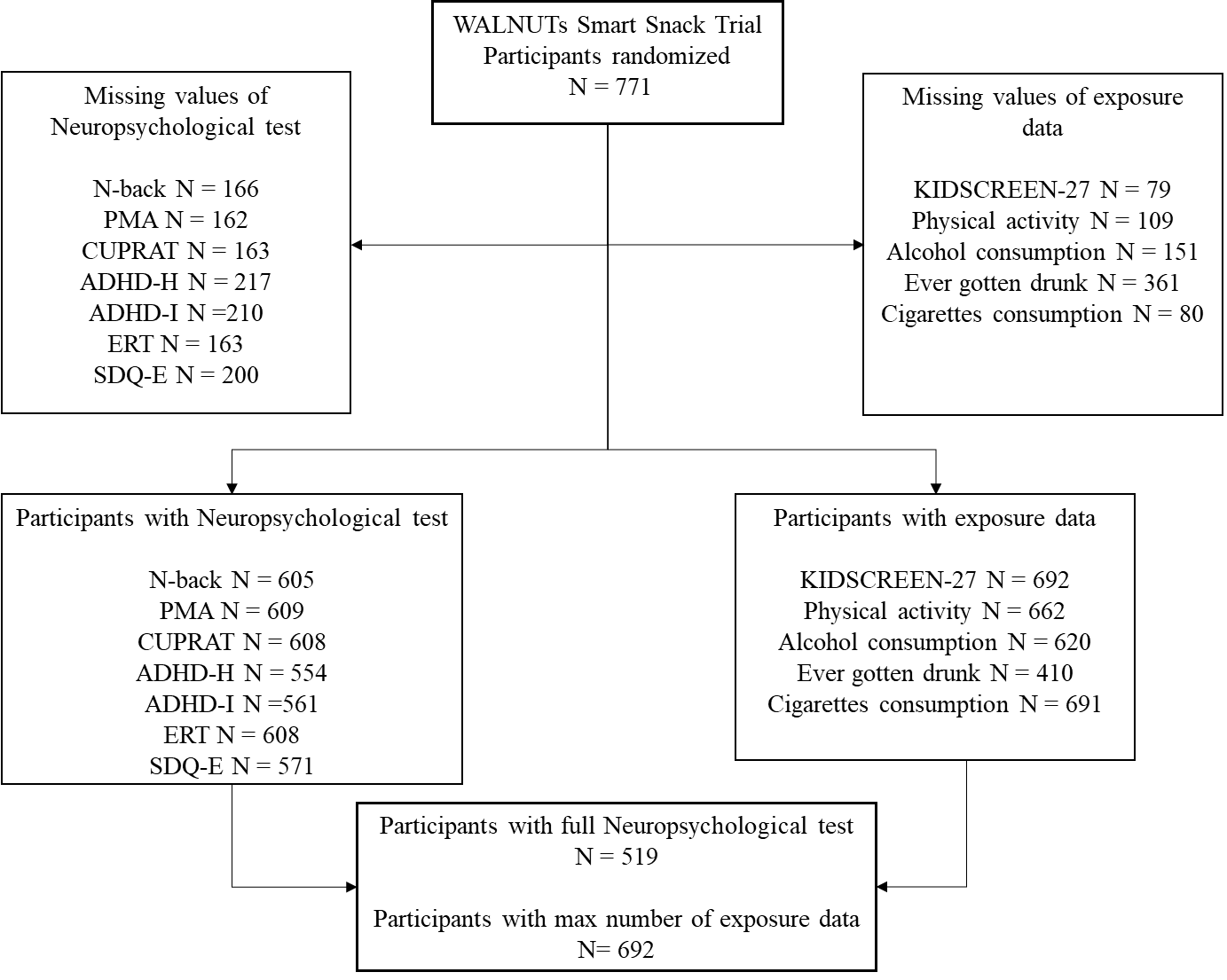


**Supplementary Figure 2**. Directed Acyclic Graph for investigating causal paths between psychological well-being, lifestyle variables, and neuropsychological functions in adolescents.


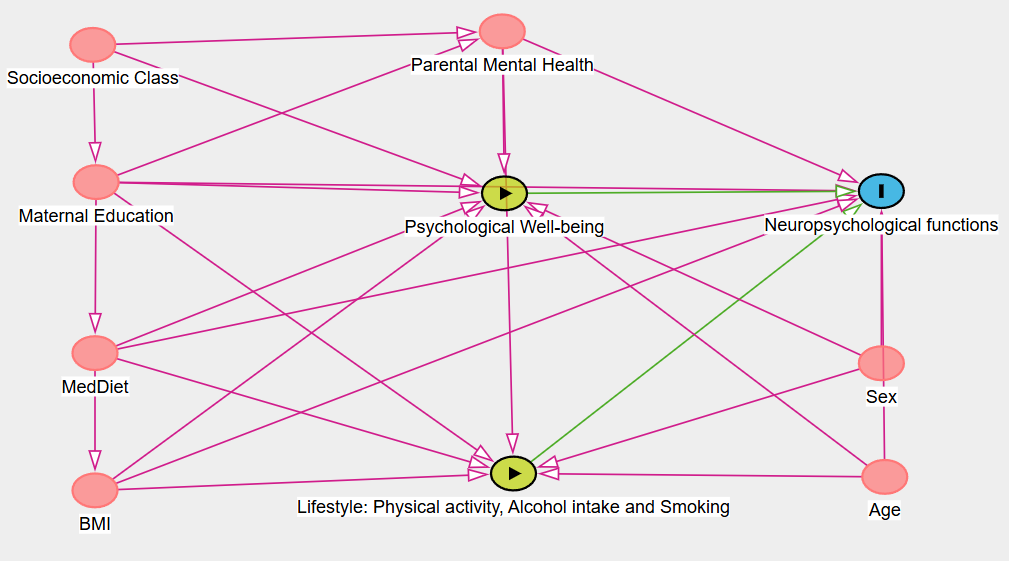


BMI body mass index.

**Supplementary Figure 3**. Scree plot for principal component analyses of neuropsychological variables.


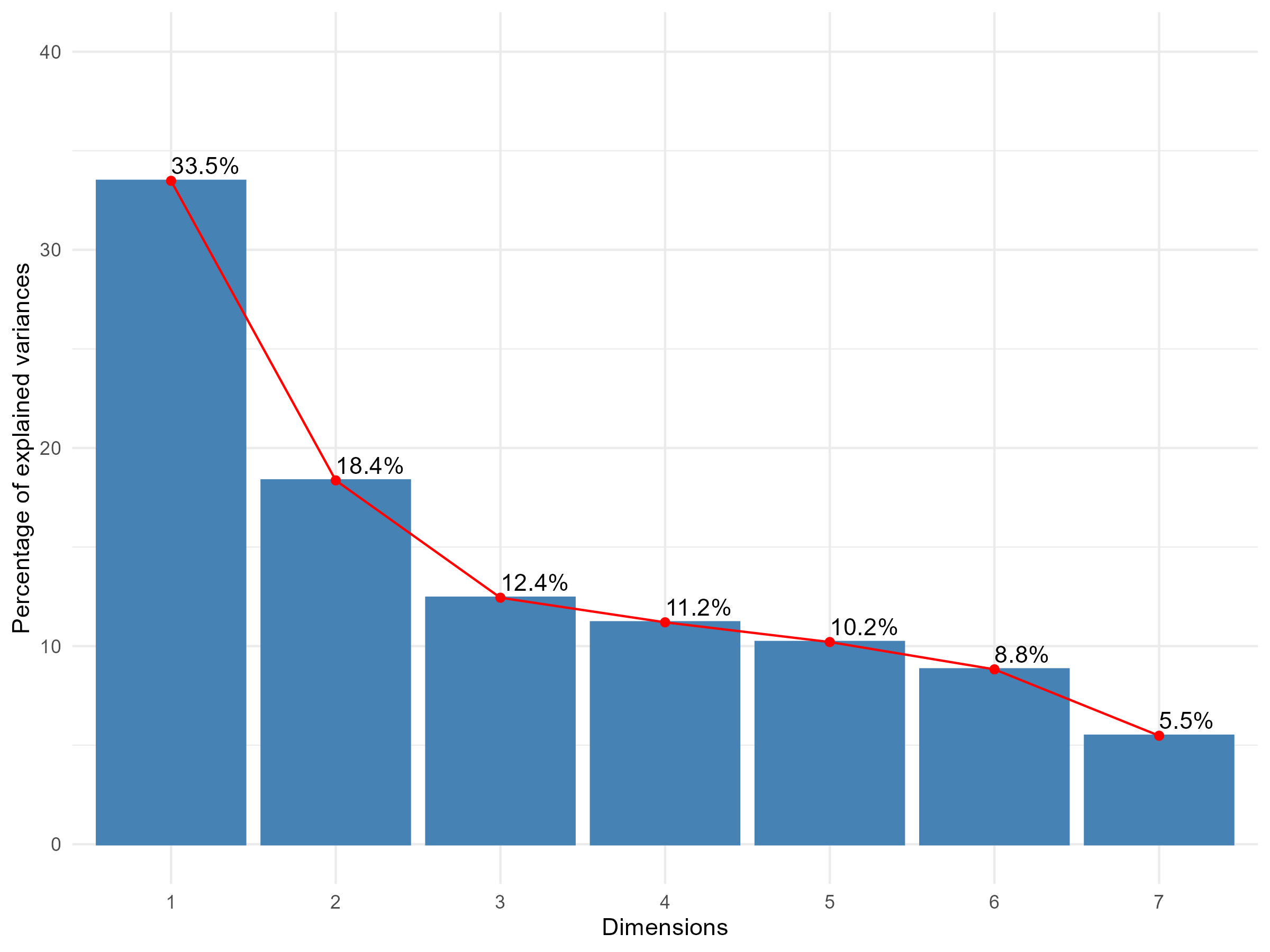


Dimensions in x-axis represents the number of principal components. PC1 and PC2 correspond to “ADHD symptoms” and “hot executive functions”, respectively.

**Supplementary Figure 4**. Variance inflation factor of independent variables of multivariate linear regression models.


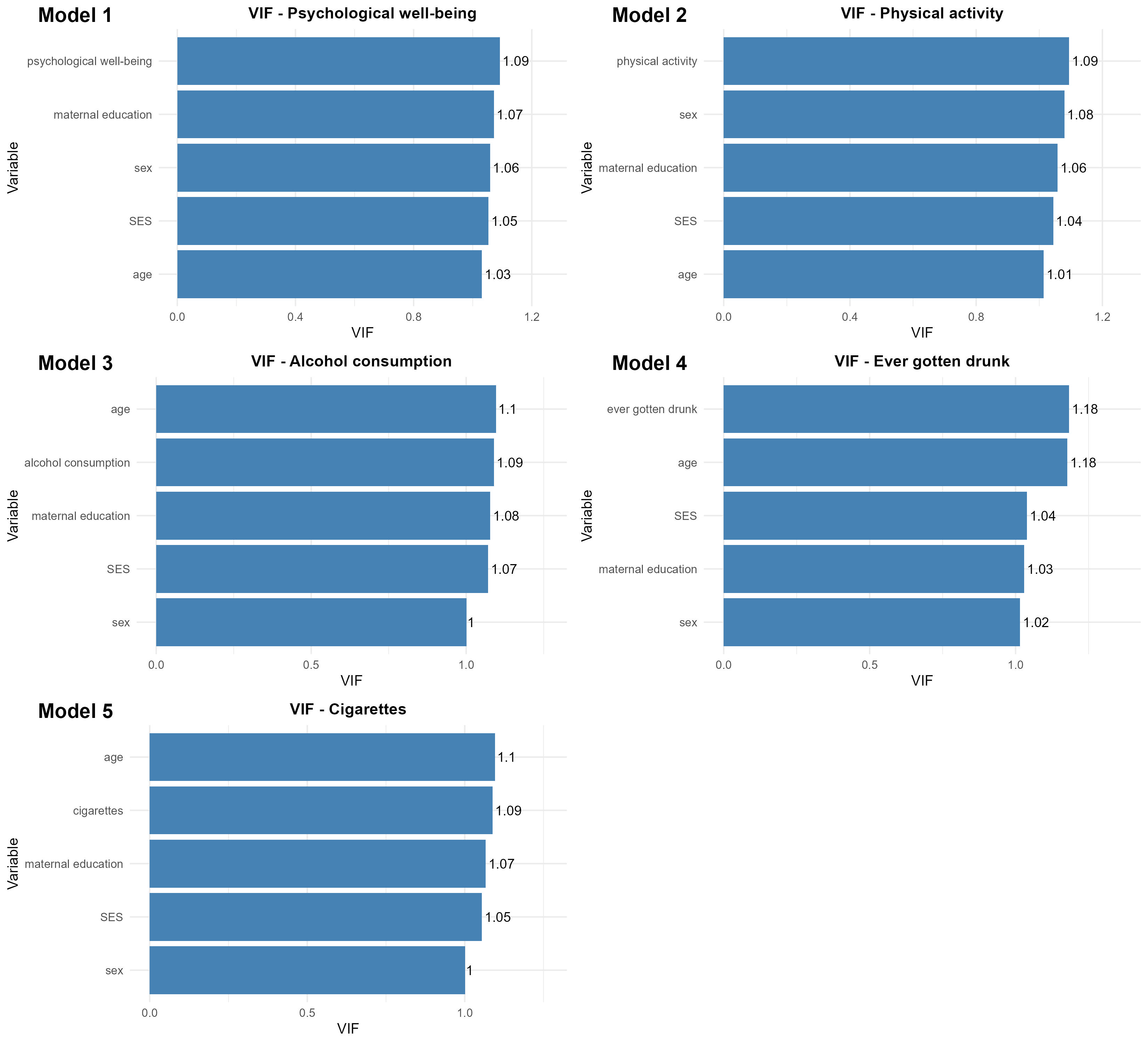


Variance Inflation Factor (VIF) of independent variables in multivariate linear regression models with dependent variables PC1 “ADHD symptoms” and PC2 “hot executive functions”, computed on the dataset used in the regressions.

**Supplementary Figure 5**. Testing linear regression assumptions for associations between psychological well-being, physical activity, alcohol and cigarette consumption with “ADHD symptoms” principal component.


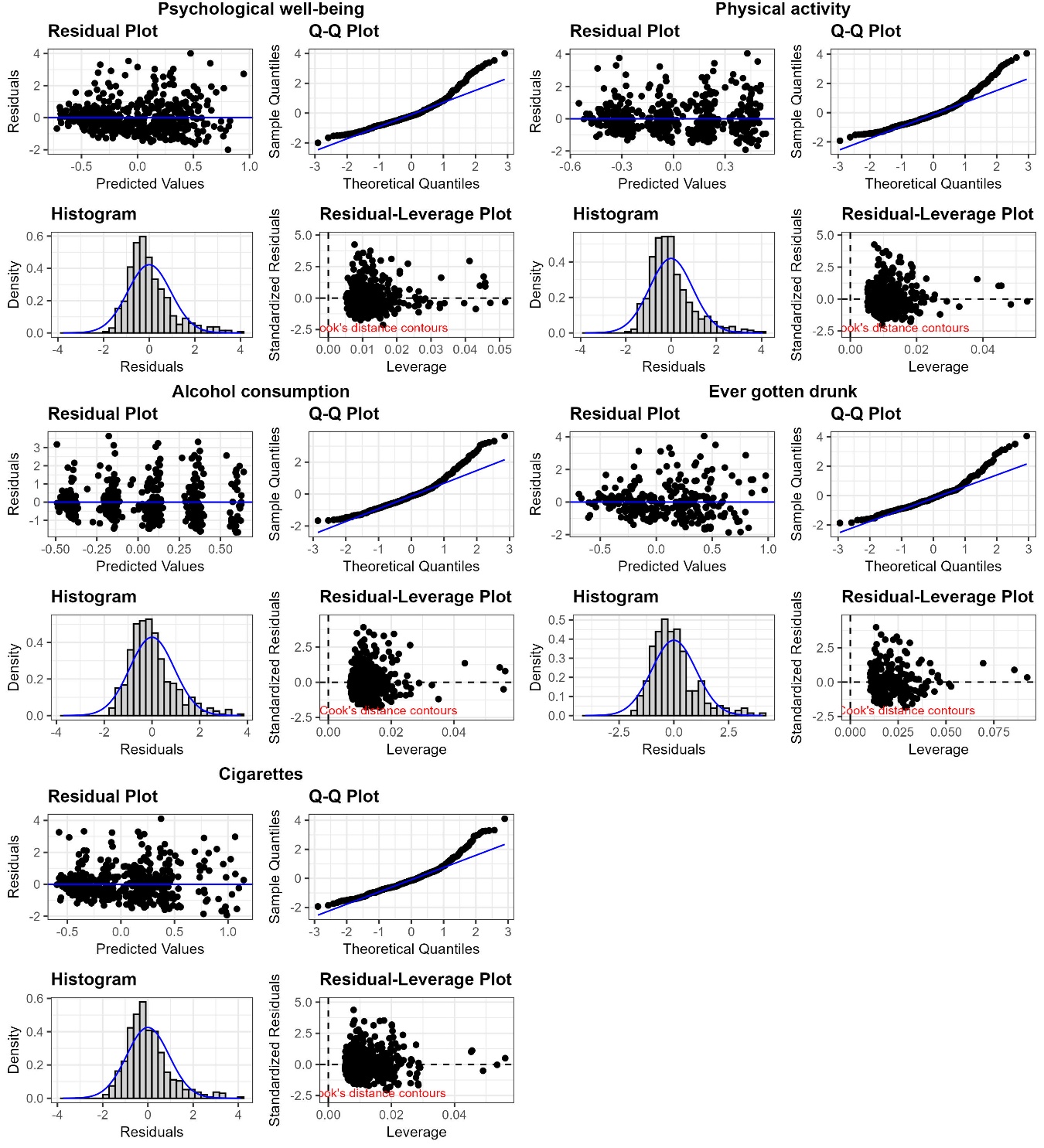


Multivariate linear regression models adjusted for exposure, sex, age, maternal education and socioeconomic status.

**Supplementary Figure 6**. Testing linear regression assumptions for associations between psychological well-being, physical activity, alcohol and cigarette consumption with “hot executive functions” principal component.


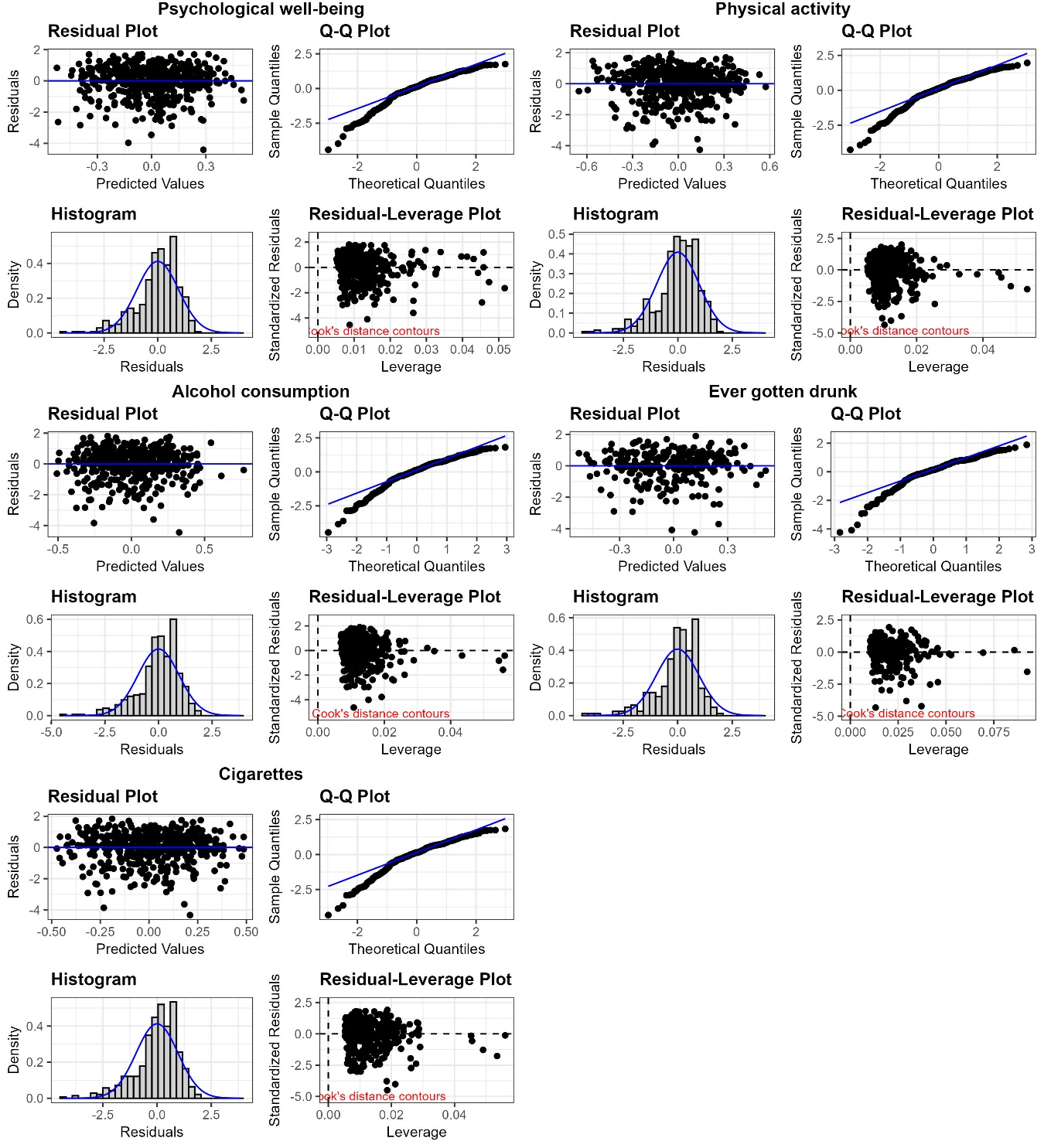


Multivariate linear regression models adjusted for exposure, sex, age, maternal education and socioeconomic status.
